# No Forcible Eviction

**Published:** 1920-01-24

**Authors:** 


### No Forcible Eviction.

LOCAL authorities are advised to make reasonable progress with the clearance of unhealthy areas before July 31, 1921, otherwise they will not be eligible for the financial grant allowed by the Ministry for such cases. At the same time the local authority should on no account actually turn people out of their present homes, defective even though these may be, unless new, or at any rate temporary, accommodation is available for them. It is well known that many historic houses are situate in some of the worst slum areas, and in the past the principle of the sacrifice of the lesser for the greater good has been applied somewhat ruthlessly. Thus we are pleased to note that the Ministry wishes "special consideration" to be given to any buildings which, "for historical, æsthetic, or other reasons, it may be in the national interest to preserve." The manual indicates that the Government evidently means to deal with the shortage of houses with the utmost despatch, in order to cope with another grave problem—that of insanitary property. So great is this urgency deemed that the Government are not prepared to subsidise remedial schemes except where "such energy and despatch is shown in their adoption and fulfilment as is demanded by the interests of the nation."

Such stout exhortations ought to stir our somewhat lethargic local officialdom into some semblance of activity, and we can only hope they may, for this question is one of paramount and really national importance.

